# The clinical trials puzzle: How network effects limit drug discovery

**DOI:** 10.1016/j.isci.2023.108361

**Published:** 2023-10-30

**Authors:** Kishore Vasan, Deisy Morselli Gysi, Albert-László Barabási

**Affiliations:** 1 Network Science Institute, Northeastern University, Boston, MA, USA; 2 Department of Statistics, Federal University of Parana, Curtiba, Brazil; 3 Department of Veteran Affairs, Boston, MA, USA; 4 Department of Medicine, Brigham and Women’s Hospital, Harvard Medical School, Boston, MA, USA; 5 Department of Data and Network Science, Central European University, Budapest, Hungary

**Keywords:** Medicine, Bioinformatics

## Abstract

The depth of knowledge offered by post-genomic medicine has carried the promise of new drugs, and cures for multiple diseases. To explore the degree to which this capability has materialized, we extract meta-data from 356,403 clinical trials spanning four decades, aiming to offer mechanistic insights into the innovation practices in drug discovery. We find that convention dominates over innovation, as over 96% of the recorded trials focus on previously tested drug targets, and the tested drugs target only 12% of the human interactome. If current patterns persist, it would take 170 years to target all druggable proteins. We uncover two network-based fundamental mechanisms that currently limit target discovery: *preferential attachment*, leading to the repeated exploration of previously targeted proteins; and *local network effects*, limiting exploration to proteins interacting with highly explored proteins. We build on these insights to develop a quantitative network-based model to enhance drug discovery in clinical trials.

## Highlights

• The rate of novel drugs has decreased since 2001, entering a drug discovery winter
• Target selection is by two processes: preferential attachment and local network effects
• A quantitative model helps unveil the mechanisms capable of boosting drug innovation

## Graphical abstract

**Figure:**
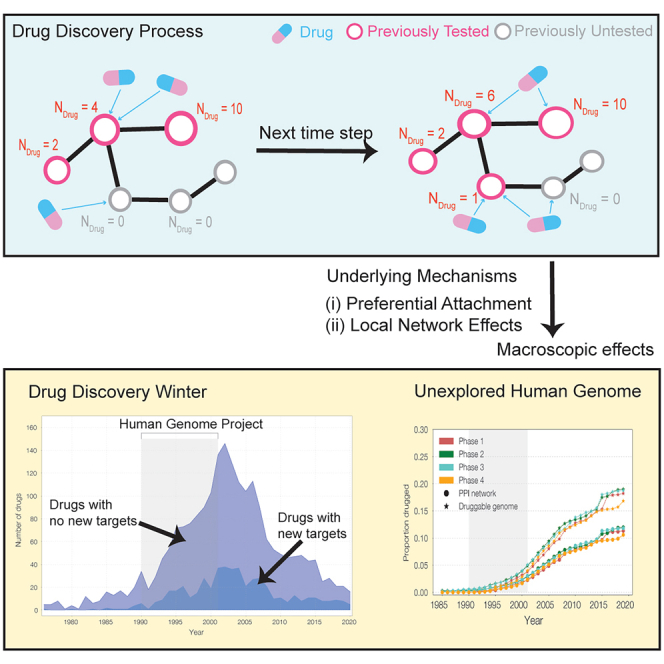

## Introduction

Prior to receiving approval by the Food and Drug Administration (FDA), a new drug must complete multiple phases of clinical trials to prove its efficacy and safety. The complete clinical trials pipeline for a single drug, from early safety testing to trials on large populations, takes on average six years,^1^ and is estimated to cost about $1 billion USD.^2^ In 2007, the FDA Act^3^ required funders to publicly post clinical trial designs and results to an online repository managed by the National Library of Medicine (NLM), increasing transparency in the drug discovery process.^4^ Despite well-documented compliance issues on reporting the results,^5^^,^^6^^,^^7^ the accumulated data offer a unique lens into the drug innovation practices,^8^ and has allowed researchers to conduct meta-analyses on disease specific trials,^9^^,^^10^ obtain key insights into equity for patients with rare diseases,^11^^,^^12^ and unveil systemic biases in patient demographics.^13^^,^^14^

The choices in clinical trials, from designing the trial protocol to selecting the patient population to testing drugs for specific diseases, have direct implications for the efficacy and equity of drugs that enter the market. While advances in genomics, machine learning,^15^^,^^16^ network medicine,^17^^,^^18^ and pharmacology^19^ present novel opportunities for drug discovery, potentially reducing the cost and time of conducting exhaustive experimental testing,^20^ they may be inadequate if the discovered knowledge about drug candidates (*in silico*) is not actively transferred to applied settings (*in vitro*), and make their way into clinical practice. Therefore, understanding the drug exploration patterns documented by clinical trials is important to improve population health.^21^^,^^22^

In this work, we offer a large-scale temporal analysis of drugs and its target’s trajectory through clinical trials by exploring the cumulative knowledge of the clinical trials database. By combining data from various sources, including investigational and approved drugs, rare and common diseases, proteins and its disease associations, we aim to understand the factors driving the discovery and exploration of new drugs and targets. We find that while the number of clinical trials continues to increase, the rate of novel drugs entering clinical trials has decreased since 2001, a puzzling effect potentially indicating a drug discovery winter. We also find that target selection is primarily driven by two distinct network-based mechanisms, preferential attachment and local network effects, leading to the over exploration of certain drugs and protein targets. Our results illustrate that we currently fail to utilize the complete therapeutic potential of the human genome, prompting us to offer a data-driven pathway to unlock its potential through the human interactome, which captures the physical interaction between targets. We build a quantitative model of drug discovery that helps unveil network effects capable of boosting the identification of novel targets.

## Results

### Curating clinical trials and drugs

We extracted the clinical trials data from the publicly available clinical trials portal (https://clinicaltrials.gov), documenting 356,403 trials from 1975 to 2020. We observe a rapid growth in the number of reported drug trials before the 2007 activation date of the FDA amendment that required all funders to publicly disclose all active clinical trials by that year (Figure 1A, vertical line), likely reflecting the sudden registration of all ongoing trials. Following 2007, an organic growth sets in, indicating compliance with public reporting of new trials.

**Figure 1:**
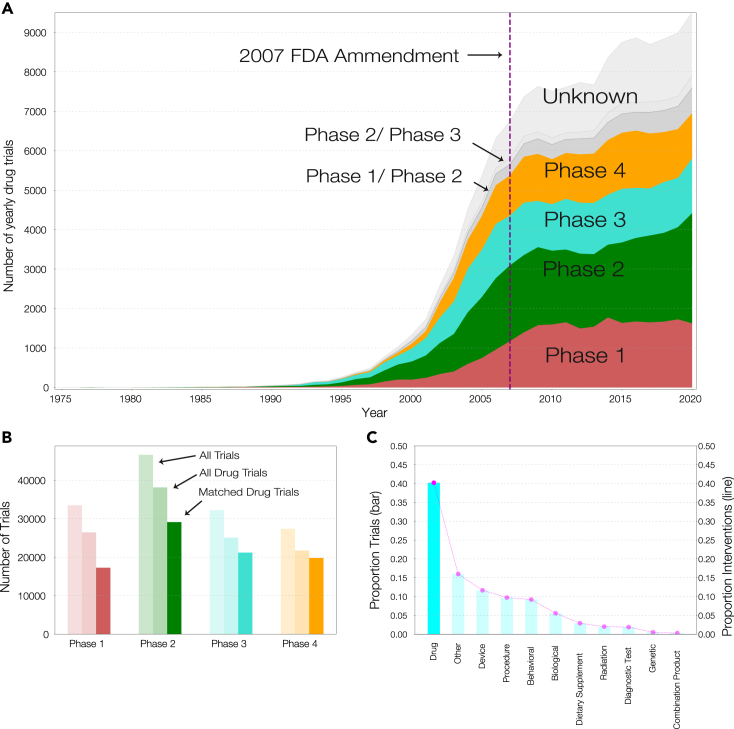
Clinical trials over time (A) Number of drug trials initiated over time. The rapid rise in clinical trials prior to 2007 is likely due to the 2007 FDA act that required all ongoing clinical trials to be registered on clinicaltrials.gov (purple line). We limit our analysis to phases 1 to 4 of clinical trials, and disregard combined phases and trials with unknown phase (gray). (B) Number of trials grouped by phase. We filter all known drug trials and match the drug interventions listed on the trials to known drugs (Supplementary Section 1.3). We show the final number of trials, grouped by phase, representing the corpus for our analysis (dark shade). (C) Proportion of trials and interventions by intervention type. Here we focus on drug trials, which represent roughly 40% of all clinical trials and 30% of all interventions.

We conducted a multi-step data standardization process to disambiguate drug names listed on trials (see STAR Methods), enabling the identification of 5,694 drugs used in 127,432 trials (89% of drug trials). A drug is designed to bind to specific proteins in the human interactome, known as primary drug targets, responsible for the desired therapeutic effect. In some cases, drugs can also indirectly bind to other proteins, referred to as secondary drug targets. Of the 5,694 identified drugs, 2,528 (44%) drugs have associations to 2,726 drug targets (both primary and secondary) and 1,442 (25%) drugs have associations to 1,842 primary targets. We consider both primary and secondary targets, but we find that our results apply even when we limit our focus on primary targets only (see STAR Methods).

Clinical trials are divided into several phases.^23^ The pre-clinical stage (Phase 0 or early Phase 1) involves small dosage of a drug on a few people for a short duration to measure treatment response, corresponding to 1,880 (1.5%) trials in our data. Phase 1 is the first full-scale human trial that includes close monitoring of treatment on a small number of patients, representing 26,207 trials (18%). Phase 2 requires 25 to 100 patients with a specific disease condition to test for drug efficacy, representing 37,784 (26%) trials. Phase 3 usually involves several hundred patients, where the experimental drugs are tested alongside other drugs to compare side effects and drug efficacy, representing 24,896 (17%) trials. Finally, Phase 4 often involves thousands of patients, aiming to gain additional knowledge on drug safety over time, interaction with various diseases, and consists of 21,632 (15%) trials. Some trials combined multiple phases such as Phase 1/Phase 2, Phase 2/Phase 3, together representing 11,381 (8%) trials in our database. Here, we focus only on drug trials in Phases 1 to 4, representing in total 110,519 (76%) trials (Figure 1B highlighted), and disregard 19,718 (13%) trials without phase information (Figure 1A, gray). Clinical trials can test multiple types of interventions, from drugs to medical devices to behavioral studies. Drugs, the most widely tested intervention, represent 40% of all trials, followed by medical devices (10%) and behavioral interventions (10%) (Figure 1C).

### Drug discovery winter

The Human Genome Project (HGP), lasting from 1990 to 2001, boosted innovation and drug exploration,^24^ as in this decade clinical trials tested 768 (30% of all) new drugs and 1,149 (42% of all) new targets (Figure 2 A shaded). Yet, beginning 2001, the exploration of new drugs has reduced. For example, between 2011 and 2020, clinical trials tested only 339 (13%) new drugs and 662 (24%) new targets (Figures 2A and S11), which, on average, corresponds to 33 new drugs and 24 new targets yearly, considerably lower compared to 99 drugs and 113 targets tested yearly in the early 2000s. Further, of the 339 new drugs that entered clinical trials, only 88 (25%) drugs have novel targets, i.e., targeting not previously targeted proteins (Figure 2A, bottom). This indicates a drug discovery winter that started around 2001 characterized by a large number of clinical trials that focus mainly on drugs that target proteins already targeted by other previously tested or approved drugs.

**Figure 2:**
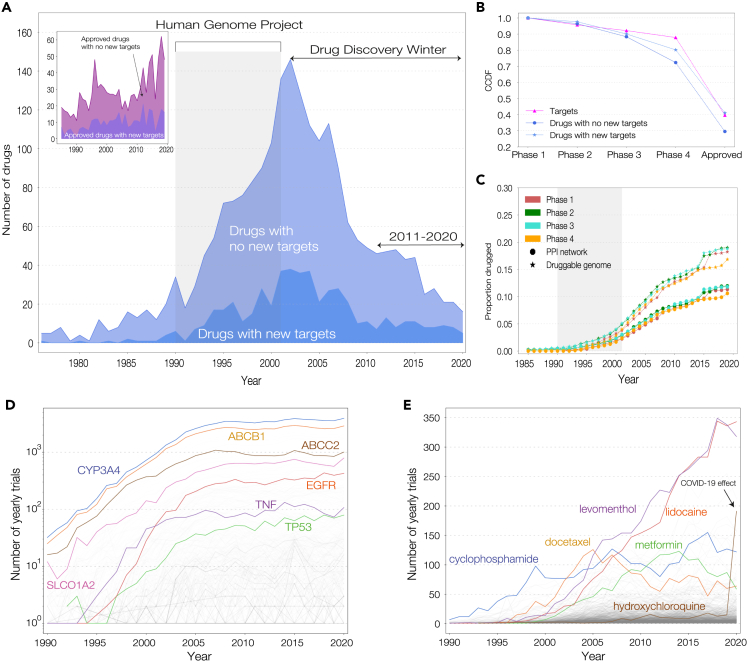
Drugs and targets tested in clinical trials (A) Number of drugs tested in clinical trials. We observe a slowdown in novel drugs tested since 2001, following the end of the Human Genome Project (HGP), signaling a drug discovery winter. For example, the number of drugs tested from 2011 to 2020 is considerably less compared to the exploration in previous decades. We also find an increasing gap between the number of approved drugs that have new targets and approved drugs with no new targets (inset). (B) Complementary cumulative distribution (CCDF) of the tested drugs and targets in each phase. We consider a protein and a drug in Phase 4 to have successfully completed Phase 1–3. The plot indicates that only a small proportion of drugs and targets in phase 4 have been approved. (C) Proportion of targets in the entire human genome in trials. We find that less than 20% of all proteins have been tested in trials. The sudden jump in number of proteins in 2015 is due to a single publication in 2015 that found 306 targets for the drug *fostamatinib* (see SI). (D) Number of yearly trials of the top targets, demonstrating the inequality of drug exploration. Some targets, like CYP3A4, ABCB1, and ABCC2 (highlighted) are the focus of multiple trials, while other targets are tested in only a few trials each year. We would like to highlight that we treat the presence of multiple drug-target associations for a single protein within an individual clinical trial as a single occurrence of a clinical trial for that specific protein. By adopting this approach, we effectively remove the number of drugs as a confounding factor when analyzing the number of yearly trials associated with a particular target. (E) Number of yearly trials for drugs. A select few drugs like levomenthol and lidocaine are tested in several trials every year, while other drugs are rarely tested. We see the impact of COVID-19 with a rapid increase in the number of trials for hydroxychloriquine.

Throughout the history of clinical trials, 956 drugs (17% of all), involving 1,340 targets (49% of all) have been approved by the FDA (Figure 2A inset). Yet, only 342 (35%) approved drugs test novel targets, indicating that drugs with established targets are more likely to receive approval.^25^ Although 1,449 (70%) drugs and 2,076 (81%) targets have reached Phase 4, only 40% of those drugs and 51% of those targets in Phase 4 targets received approval (Figure 2B). We also find that, on average a drug experiences a 3-year lag for approval after successfully completing Phase 3 clinical trials capturing the slow approval period, despite standard clinical development times^26^ (Figure S15). Taken together, we find that clinical trials have tested only 12% of all human proteins and 22% of all druggable proteins^27^ (Figure 2C). We estimate that if the current exploration patterns persist, it will likely lead to the exploration of 2,477 (13% of all) proteins by 2025, and following this rate, it would take 170 years to test all 10,648 druggable proteins (see STAR Methods).

### Previously tested proteins are repeatedly selected for future trials

Clinical trials tend to focus on a small number of previously tested proteins, leading to an uneven approach to drug discovery (Figures 2 and S12). For example, we find that CYP3A4, ABCB1, ABCC2, SLCO1A2, proteins associated with the drug metabolism and transportation,^28^ are involved in 72,884 (66% of all) trials, while EGFR, TNF, TP53, proteins associated to auto-immune diseases and several neoplasms, are involved in 8,396 (8% of all) trials (Figure 2D). Similarly, we find lidocaine, levomenthol, drugs that serve as anesthetics, to be over-represented in trials (Figure 2E). The COVID-19 pandemic had also a detectable impact on trial activity: hydroxychloroquine, a dormant drug which had a few clinical trials for over a decade, experienced a rapid increase in the number of trials in 2020^29^ (Figure 2E).

A consequence of this uneven drug-target exploration is that only a small number of trials focus on new targets, new drugs, and new target combinations (Figures 3A–3C). The majority of the trials (50%) involve only previously approved drugs, while 11% of the trials test a combination of approved and experimental drugs (Figure 3D). Seeking to find the patterns responsible for this over-exploration of previously targeted proteins, we measured to what degree targets that received more attention in the past are tested in subsequent years. We find that the number of drugs that target a specific protein, $N_{drug}\left(t\right)$, is well approximated by a growth rate following, $N_{drug}\left(t\right)\propto N_{drug}^{\gamma }\left(t-1\right)$, where γ is a scaling exponent (Figure S13; $\gamma_{2000}=1.2$, $\gamma_{2010}=1.1$, $\gamma_{2020}=0.9$). This pattern, known as preferential attachment, is known to be responsible for the emergence of network hubs in network science^30^^,^^31^ and quantifies the degree to which previously tested proteins have a cumulative advantage over other proteins.

**Figure 3:**
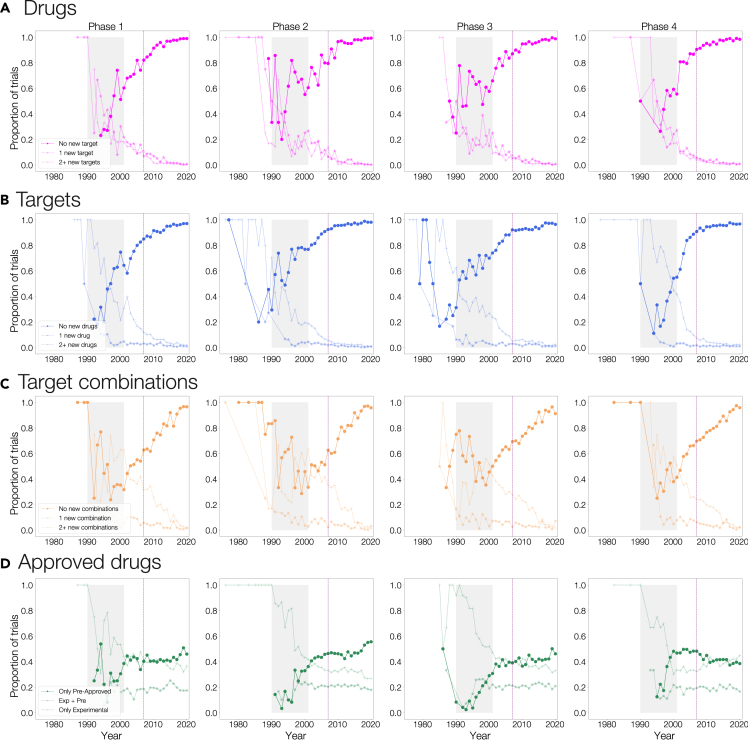
Novelty in clinical trials For each phase we identified the first time a drug, a target, or a target combination was first tested. We then trace the proportion of trials in each year for each phase that focus on (A) new targets (B) new drugs, and (C) new target combinations. Across (A–C), we observe a rapid rise in trials with new targets, coinciding with the completion of the HGP (shaded). Starting in 2005, only a minimal percentage of trials across various phases are dedicated to exploring novel targets, drugs, and combinations. (D) We also observe that close to half of the trials each year test previously approved drugs, indicating high interest in drug repurposing. This may partly be motivated by patent laws that force the patent owners to find new uses for the drug compound. As a consequence, we find growing inequality, where a select list of targets of approved drugs is repeatedly in clinical trials, thus preventing broad exploration of the human genome.

### The role of human interactome in drug exploration

Some diseases can be treated by inhibiting the disease associated proteins, but most often the effective drugs target proteins that are in the network vicinity of known disease proteins.^32^ Indeed, most drugs act by modulating the activity of the sub-cellular web known as the human interactome,^33^ captured by experimentally detected protein-protein interactions (PPI). As pharmaceutical scientists leverage this network topology during the development of small molecules, it prompts us to inquire whether we can harness the power of the interactome to explain the underlying patterns that define target discovery and exploration. To answer this question, we first mapped the 2,726 drug targets explored in clinical trials into the interactome, finding that 1,260 (92% of all) experimental drugs target at least one protein that has been previously targeted by another approved drug, in line with Figures 2 and 3. However, when focusing on the proteins not targeted by previously approved drugs, we find that 891 (76%) of them interact with at least one protein that is targeted by an approved drug, while 274 (23%) are two steps away from the target of an approved drug. This local network-based clustering of experimental and approved drugs is absent if we randomly select the drug targets (see STAR Methods).

We also find that proteins located farther from approved and experimental targets are rarely selected as a drug-target, even if they have multiple disease associations and are known to be druggable. In other words, we find a strong preference for targeting proteins that are embedded in local network neighborhoods with multiple explored targets (Figure S22). This means that a protein that interacts with other proteins that are the subject of multiple clinical trials for experimental or approved drugs is more likely to be selected as a new drug-target compared to a protein located in an unexplored network neighborhood. This suggests that the protein-protein interaction network captures and potentially drives drug discovery and exploration.^34^

To unlock the impact of the observed network effects, we examine the likelihood of a protein to be selected as a drug-target in a future clinical trial using a Generalized Linear Mixed Model (GLMM). The GLMM model considers as input four features of each target: (1) disease associations, (2) number of approved drugs targeting it, (3) number of clinical trials it was involved in, and (4) number of experimental drugs targeting it (see STAR Methods). The model is used for inferential purposes, offering as output several insights on the mechanisms governing new drug-target exploration (Tables S3–S5):

1. Disease associated proteins are two times more likely to be in a clinical trial compared to proteins with no disease associations (OR: 2.2 [CI:1.6, 3.2], p < 0.05).

2. Proteins experience increased likelihood of becoming the target of a new drug when they are already targeted by multiple approved drugs (OR: 3.7 [CI: 3.6, 3.9], p < 0.01), multiple experimental drugs (OR: 2.7 [CI: 2.6, 2.8], p < 0.01), or are the subject of multiple trials (OR: 1.47 [CI: 1.45, 1.49], p < 0.01).

3. Previously untargeted proteins are more likely to be selected if they interact with proteins associated with multiple approved drugs (OR: 1.01 [CI: 0.99, 1.04]), multiple trials (OR: 1.03 [CI: 1.01, 1.04], p < 0.01), or multiple experimental drugs (OR: 1.05 [CI: 1.03, 1.07], p < 0.01).

These findings establish two fundamental mechanisms that drive drug exploration.

(1) *Preferential attachment:* The future attractiveness of a protein as a drug candidate increases as more drugs target it and more trials focus on it (increased clinical exposure). For example, for a protein that is already targeted by ten drugs, its odds of being the target of a new drug increase 8-fold, compared to a protein not targeted by a drug.
(2) *Local network effects*: Previously untargeted proteins located in network neighborhoods with high exploration patterns (containing multiple drug targets and clinical trials) are more likely to be selected as new drug target compared to proteins located in network neighborhoods with fewer clinical trials and drugs.

### Modeling choices in drug discovery

We build on the insights (1) and (2) to introduce a network model that aims to quantitatively recreate the observed patterns in drug exploration, and helps us understand how to accelerate drug discovery by exploring a wider set of druggable candidates. We begin by creating a timeline of drug discovery, accounting for the precise dates when targets became associated with drugs (Figure 4A ). Using the proteins (nodes) and its interactions (links) in the PPI network as the underlying space of possible exploration, we model drug discovery through two parameters: The parameter *p* represents the probability that a previously tested protein is selected again for clinical trials. Hence, for p=0, we model the scenario where we always choose untargeted proteins, while for p=1 we always select previously tested proteins as targets. The second parameter, *q*, represents the probability that we choose an untargeted protein that is part of an explored neighborhood, driven by local network search (Figure 4B). Hence, for q=0, we always select proteins from unexplored neighborhoods, while for q=1 we select proteins from previously explored neighborhoods. Finally, to account for preferential attachment in target selection, a previously tested protein is selected again as a target proportionally to the number of drugs that have targeted it in the previous years, $P\left(N_{drug}\left(t\right)\right)\propto P\left(N_{drug}\left(t-1\right)\right)$ (Figure S13).

**Figure 4:**
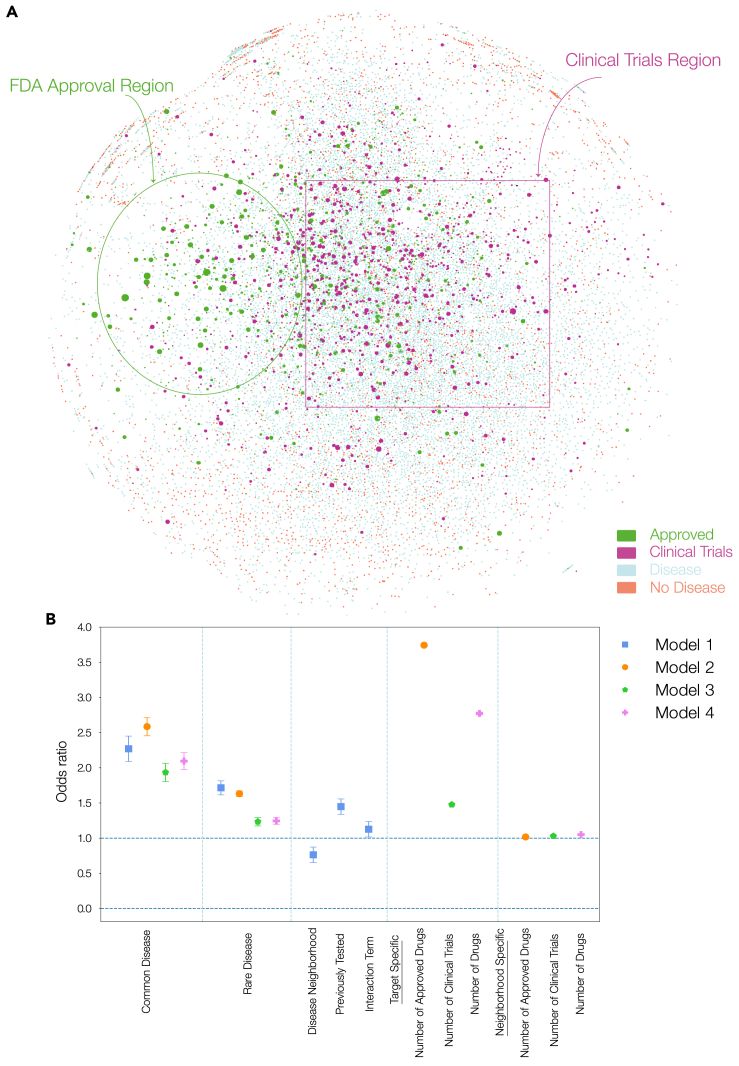
Modeling mechanisms of drug-target discovery (A) The exploration of the protein-protein interaction (PPI) network, where new proteins are selected as targets for drugs in clinical trials. For time $t_{0}$, we calculate the number of drugs that previously targeted each protein in the network, $N_{Drug}\left(t_{0}\right)$. At the timestep $t_{0}$, new drugs are introduced in clinical trials for testing. We identify the targets of these drugs at time of trial, represented using arrows, and update the number of drugs for proteins at the next time step, $N_{Drug}\left(t_{1}\right)$. Similarly, we identify the drugs introduced at time $t_{1}$ and its targets and update the number of drugs that target a protein at time $t_{2}$. The temporal characteristics of each protein allow us to capture the drug discovery process in clinical trials. (B) Network model. We consider the network at time step, $t_{0}$, using the above described process and group proteins into three categories: (i) proteins that were previously tested, (ii) proteins connected to a previously tested protein, and (iii) proteins that are not connected to a previously tested protein. With probability *p*, we select a previously tested protein, while with probability *q* we select a protein connected to a previously tested protein, and with probability 1−q, we select a protein not connected to a previously tested protein. When choosing a previously tested protein, we sample proteins proportional to the number of drugs that have previously targeted it, $P\left(N_{Drug}\right)$, representing preferential attachment. In the network simulations, we select m(t) proteins (calculated from data) and update the network at the end of each time step. We describe one version of the simulation where parameters n=3,p=0.5,q=0.8 are used to select the proteins *B*, *C*, and *D* at next time step $t_{1}$. (C) The search space of exploration. We measure the number of targets that are tested in the simulations as a function of the parameters *p* and *q*. The circles indicate the empirical choices for different years (2010, 2015, and 2020). We show the distribution of number of drugs per target obtained under the three different exploration strategies: (D) Preferential attachment (PA) (p=0.95,q=0.95). (E) Random (R) (p=0.5,q=0.95) and (F) network search (NS) (p=0.05,q=0.95).

The advantage of the proposed model is that we can explicitly extract the parameters *p* and *q* from the clinical trials data (Figure 4C). For example, in 2010, 295 proteins were tested in clinical trials, of which 244 (82%) were tested in previous clinical trials, and we find that of the 51 previously untargeted proteins, 45 (88%) interact with a previously tested protein, hence p=0.82 and q=0.88. We find that the empirically obtained (*p*,*q*) parameters are remarkably stable over time, indicating that previously tested proteins are in each year preferred at high rates ($p_{2010}=0.82$, $p_{2015}=0.78$, $p_{2020}=0.91$; Figure S21). We also find that among the untargeted proteins, those interacting with other previously tested proteins are more likely to be selected ($q_{2010}=0.88$, $q_{2015}=0.92$, $q_{2020}=0.87$), allowing us to quantify the stable patterns characterizing drug discovery (Figure S22). As Figure 4C shows, the empirically observed patterns are stable in the high (*p*, *q*) regime, with a slight shift over time to higher values of *p* and *q*, confirming an increasing trend to explore previously tested targets.

We find that for the observed ($p^{*}$, $q^{*}$) values, the network model accurately reproduces the distribution of number of drugs per target (Figure 4D; KS-distance: 0.06; p < 0.01). The model also allows us to test the relative importance of its building blocks. For example, if we remove the preferential selection of targets, the model fails to capture the drug exploration patterns (Figure S26), confirming that preferential attachment (PA) is a key ingredient of the current drug exploration strategy. The model also unveils the imperfections of the current target selection patterns: the PA strategy, which redirects attention and resources to previously tested proteins, only tests 21 new targets yearly on average. As a consequence, the same protein is explored as a target for a total of 175 (17% of all) drugs (GINI=0.65 t/b), acting as a hub of drug discovery. Overall, the current strategy, by repeatedly targeting previously tested targets, fails to take advantage of the broader potential of the interactome to unveil potential novel targets. To validate the model, we quantified its ability to predict drug candidates for three autoimmune diseases—rheumatoid arthritis (RA), Crohn’s disease (CD), and asthma (see STAR Methods). We find that the model accurately predicted novel candidates for these diseases with 70% accuracy (Figure S28). Further, we validated the predicted proteins through an extensive literature search, finding them to be biologically relevant (Table S6). For example, the model identified protein *NLRP3* as a potential drug candidate for RA, which has been shown to reduce RA-induced inflammation in animal models.^35^ These results demonstrate that a network strategy can be a useful mechanism to drive exploration toward proteins in druggable parts of the network.

Finally, we want to exploit the predictive power of the network model to explore how to incentivize a wider exploration of human interactome as potential targets. For this, we examine two alternative exploration strategies: (1) random (R) strategy, when the newly tested proteins are randomly selected (p=0.5); (2) network search (NS) strategy, when untargeted proteins interacting with previously targeted proteins are preferred (p=0.05). In each case we keep q=0.95, as indicated by the empirical data.

We find that the random (R) strategy selects more drug targets than currently tested (as captured by the PA strategy) (2,655 vs. 1,121), offering an opportunity to deviate from the current distribution of number of drugs per target (Figure 4E, KS-distance: 0.22, p < 0.01). Despite the randomness of the strategy, the same protein is selected as a target for 110 (11% of all) drugs (GINI=0.35), indicating that the R strategy also focuses repeatedly on a few network hubs, a pattern similar to the one observed in the PA strategy (175). Overall, the R strategy tests more targets than PA but still results in an over-exploration of a few proteins, and hence offers minimal improvements compared to PA (Figure S27).

In contrast, we find that the network search (NS) strategy generates statistically different distribution of number of drugs per target (Figure 4F; KS-distance: 0.37; p < 0.01). Most importantly, the strategy selected 4,055 targets, a 3-fold increase in the number of selected targets compared to the PA strategy (1,121). Of those 4,055, we find that 3,922 (96%) are new targets. Further, the NS strategy selects the same protein as a target for a maximum of 10 (1% of all) drugs (GINI:0.06), significantly lower compared to the R (110) or PA (175) strategies.

Overall, our results indicate that the current practice (PA) is inefficient in terms of exploring the human interactome, focusing most resources on a small number of highly explored protein targets. In contrast, a network search approach can improve the total number of tested targets by preventing the emergence of protein hubs in drug discovery and also attract attention to potential drug candidates, ultimately resulting in a wider exploration of the human interactome. These results suggest that policy changes, such as prioritizing the approval of drugs with novel targets or targeted funding from the National Institutes of Health (NIH) toward the exploration of novel targets, could help augment existing innovation practices and significantly enhance drug discovery by re-focusing resources on a wider range of novel targets while maintaining accuracy.

## Discussion

A scientist’s choice of an idea to pursue is influenced by a combination of the project novelty and its potential research impact.^36^^,^^37^ Similarly, a pharmaceutical company’s choice of a target for a new drug is influenced by its potential market value and the likelihood that the drug succeeds in clinical trials.^38^ However, the high attrition rates of drugs in clinical trials,^39^ difficulties with patent licensing,^40^ and the growing cost of developing new molecules^41^ have led to a risk-averse approach to drug discovery characterized by “small bets, big wins.”^25^ While this strategy, resulting in the creation of multiple drugs within the same therapeutic class,^42^ increases competition and reduces drug prices,^43^^,^^44^ it takes away resources from the exploration of novel drugs and targets,^45^ encouraging incremental innovation and hindering progress for population health.

Our analysis of clinical trials data shows that the highest growth in drug exploration was between 1990 and 2001, likely driven by the advent of the Human Genome Project (HGP). However, in the following two decades, there was a decrease in the incentive to test novel drugs, and a disproportionate focus on approved drugs (61% of all trials). This allocation of resources ultimately slows the discovery of novel therapies. Further, drug discovery in clinical trials often prioritize previously tested proteins (preferential attachment) and proteins connected to previously tested proteins (network effect), neglecting proteins in under-explored regions of the network, even if they have disease associations and are verified as druggable targets. To optimize target exploration in druggable regions of the network and improve the number of tested targets, it may be beneficial to reduce the emphasis on previously tested proteins and adopt a network-based search for drug candidates.

It is important to acknowledge that designing a new small molecule that engages with a specific protein may be challenging despite the fact that the protein may be considered a druggable candidate. These factors encompass limitations in experimentation, such as the absence of suitable animal models, economic constraints and market dynamics, and the inherent complexities and challenges associated with discovering effective treatments. To gain a more comprehensive understanding of target prioritization, it is important to integrate network-based strategies with other relevant data sources, such as genomic information, phenotypic data, and comprehensive analysis of clinical outputs obtained from both successful and failed trials. Regrettably, the systematic reporting of such attributes by pharmaceutical companies is currently lacking.^5^^,^^6^^,^^7^ By embracing these recommendations and actively pursuing an integrative approach, we can foster a more robust and effective drug discovery process. This, in turn, will pave the way for the development of innovative pharmaceutical interventions that address unmet medical needs, ultimately benefiting patients and society as a whole.

## STAR★Methods

### Key resources table

**Table:** 

REAGENT or RESOURCE	SOURCE	IDENTIFIER
Drugs and Targets	DrugBank	DrugBank^46^
Clinical trials data	ClinicalTrials.gov	https://clinicaltrials.gov
Druggable targets	DG-IDB	www.dgidb.org, ref. Freshour et al ^27^
Protein-protein interaction network	PPI	Ref. Gysi^47^
Disease data	DisGeNet	Ref. Piñero et al.^48^
Drug approval data	FDA	https://www.fda.gov/drugs
Common and rare diseases	Orphanet	https://www.orphadata.com/, ref. Piñero et al.^48^
Target discovery data	PubMed	Ref. Gates et al.^24^

### Resource availability

#### Lead contact

Requests for further information, resources, and reagents should be directed to and will be fulfilled by the lead contact, Albert-Laszlo Barabasi (a.barabasi@northeastern.edu).

#### Materials availability

This study did not create new unique reagents.

#### Data and code availability

• This paper analyzes existing, publicly available data.
• The data and code to replicate the analysis is available at https://github.com/Barabasi-Lab/clinical_trials.
• Any additional information required to reanalyze the data reported in this paper is available from the lead contact upon request.

### Experimental model and study participant details

#### Constructing the PPI network

The proteins in the cell of an organism are known to have biological interactions with other proteins in neighboring cells. This relationship between proteins can be mapped to represent a network of genes and its interactions, a well-studied mechanism in network medicine.^47^ The protein interaction network comprises 18,508 nodes (proteins) and 332,646 edges (interactions).

We create a timeline of PPI network exploration by considering the temporal variable change of multiple protein related parameters. That is, we consider the innovation outlook of the target based on the information available at time *t*, to model the likelihood that a target will be selected at time t+1, allowing us to measure the dynamics of network visibility.

### Method details

#### Data collection and curation

##### Drugs and targets

Data about experimental and validated drugs is provided by DrugBank.^46^ DrugBank is a web-enabled database containing comprehensive molecular information about drugs, their mechanisms, their interactions and their targets and is publicly accessible using an API key at (www.drugbank.ca). The January 2021 release of this database gives us a total list of 14,315 drugs, of which 7,755 drugs are associated with 4,265 targets.

Each drug-target map has a representative publication verifying the association, representing 51,839 publications. We then use the year of publication of each paper to recreate the temporal discovery process of each drug and its target associations. We combine this information with the drug trial year allowing us to accurately identify targets that were tested in each year.

##### Clinical trials data

The contents of all listed 356,403 clinical trials was downloaded on November 1, 2020 from (https://clinicaltrials.gov). All of the studies are grouped using NCT id which serves as the identifier for each trial (Figure S8). Every trial contains information about the date of trial (Figure S6), type of trial (e.g., intervention, observational), its associated phase (e.g., Phase 1, Phase 2), status (e.g., completed, recruiting) (Figure S7), a list of conditions (e.g., asthma, rheumatoid arthritis), a list of interventions (if applicable, e.g., budenoside, inhaler) and its associated types (e.g., drug, medical device). We then filter all trials that have a “drug” type associated with any of its listed interventions. This gives a subset of 146,314 trials.

##### Clinical trials drug data curation

The listed drug names part of clinical trials are not standardized, and presents an issue to accurately identify drug exploration. For example, the drug ‘lepirudin’ may be refered to as ‘lepirudin recombinant’, ‘hirudin variant-1’ or even its associated brand name ’Refludan’. As a result, we find a total list of 94,615 interventions in the clinical much higher than the number of drugs identified by DrugBank. To standardize the drug names, we conduct a multi-step matching process. First, we map the intervention names to the direct name on drug bank, giving us a total of 103,398 (70.6% of all drug trials) trials and 4,458 drugs. Next, we map the intervention names to the drug synonyms provided by DrugBank allowing us to map an additional 7,698 trials. We also connect the drug names to the official drug product names allowing us to map another 14,759 trials. We also map intervention names with the wikipedia names of drugs providing additional drug maps for 500 drugs. Finally, we map the drugs names with a fuzzy match with drug names, providing mapping for another 1,077 trials. At the end of this methodology, we are left with 127,432 trials (87.6% of all) and 5,694 drugs. We also control for placebo drugs in trials by searching for the term ’placebo’ in the intervention names. We thus remove 1,171 trials on 590 drugs from our analysis.

The data curation steps then reveal 127,432 drug trials for 5,694 drugs and 2,726 targets, representing the final data used in the analysis.

##### Druggable genes

The list of druggable genes is curated by a large-scale crowdsourcing effort by incorporating multiple data sources (e.g., Gene Ontology, OncoKB, PharmGKB).^27^ The data is publicly available for free download from DG-IDB(www.dgidb.org) The November 2020 version of the data update was extracted for our analysis which contains 10,648 druggable human proteins. It is important to note that the finding of a drug-gene interaction as potentially druggable does not necessitate the ineffectiveness (or the lack thereof) for a drug to interact with other genes in different regions.

##### Protein-protein interaction network

The proteins in the cell of an organism are known to have biological interactions with other proteins in neighboring cells. This relationship between proteins can be mapped to represent a network of genes and its interactions, a well-studied mechanism in network medicine.^47^ The protein interaction network comprises 18,508 nodes (proteins) and 332,646 edges (interactions).

##### Experimentally validated PPI network

We conduct the analysis using the experimentally validated protein interactions, a network comprising 8,876 proteins and 61,985 interactions. We find the similar result as above, targets of experimental drugs are enriched in the region of proteins that target approved drugs (p<0.001; Figure S25), verifying that the network processes are not driven by potential selection biases of the PPI network.

##### Drug approval data

The data regarding drugs and its approval is provided by the Food and Drug Administration (FDA), publicly available at https://www.fda.gov/drugs/development-approval-process-drugs/drug-approvals-and-databases. The entire corpus was extracted in December 2020 that contains 1,002 approved drugs. After matching the FDA data with clinical trials, we found 911 drugs, representing 90% of all approved drugs.

##### Disease data

The data about disease associations were extracted from DisGeNet.^48^ We find 15,474 genes associated with 19,620 diseases. Since the data also lists the corresponding publication reference that discovered the disease association, we map the publication (PubMed) id with the year of publication to identify the specific year that the gene was found to be associated with a disease, allowing us to accurately recreate the exploration patterns (Figure S2).

The clinical trials data also contains the disease condition of the trial (e.g., hypertension). However, the disease names are not standardized. To address this issue, we use the same multi-step matching process used to curate drug data to match the disease of each trial to the curated disease data on DisGeNet.^48^ Specifically, we use string matching, fuzzy matching, and cosine similarity. We find that the top 25 diseases collectively account for 40% of all clinical trials (Figure S3).

##### Common and rare diseases

Information about common and rare diseases were extracted from *Orphanet: an online rare disease and orphan drug database* (https://www.orphadata.com/). The data are indexed via ORPHAcode that links diseases to associated genes, along with information about the association like causative, modifier, susceptibility. We then map these diseases with the DisGeNet^48^ data through Mesh ID to identify gene associations with rare and common diseases (Figure S4). After mapping, we find 29,001 common diseases associated with 15,339 genes and 1,169 rare diseases associated with 9,152 genes. The data is free to download from http://www.orpha.net. Accessed on September 2021.

##### Timeline of protein discovery and interactions

The data regarding the year of discovery of proteins and its interactions is collected by parsing 702,320 publications from the PubMed database^24^ (Figure S5). This data allows us to recreate the temporal PPI network, accounting for the precise time a protein and its interactions were discovered.

#### Selecting primary targets

Targets associated with drugs may have multiple mechanisms of action, such as, inhibitor, binder, activator, blocker, but for some drug-target associations the mechanisms of action may be unknown. The subset of targets with unknown interactions are referred to as ’off-target’ genes and those with known interactions as ’primary-target’ genes. In our data, we find that 98,139 (85% of mapped) trials featuring 1,442 (57% of drugs with targets) and 928 (34% of all) primary targets. To consider only the subset of primary targets, we must disregard more than 40% of the drugs in trials and 65% of all targets, a large proportion of lost information.

Yet, considering both primary and off-target proteins for analysis may be important to map the space of drug exploration. For example, a drug capable of modifying the activity of an off-target may provide repurposing opportunities for that drug,^49^ and may initiate future exploration of that protein. Hence, we consider both primary and off-target proteins as part of the explored proteome. We provide the results in the supplementary text when we only consider primary targets, and find that the main findings do not change (Figure S19; Table S4).

#### Fostamatinib outlier

We consider the year of drug-target association to build the target exploration in clinical trials. We do this byextracting metadata from PubMed of publications that provide verification for target associations for drugs. As we note in main text, clinical trials experience a sudden jump in exploration, attributed to the drug *fostamatinib*, that was in trial in 2015 and found its approval in 2018. In 2015, the publication titled, "*In vitro pharmacological profiling of R406 identifies molecular targets underlying the clinical effects of fostamatinib*",^50^ claimed 306 target associations for the drug *fostamatinib*. Indeed, it is very unlikely for publications to claim associations for several hundred targets (Figure S10). We remove this publication from our data in the subsequent analysis.

#### Quantifying uneven target and drug exploration

The lack of novelty in drug trials leads to repeated exploration of previously tested targets. We look at the inequality in target and drug selection using the gini coefficient, where 0 represents complete equality and 1 indicates that all trials test a single target or drug. We find a growing inequality for targets ($Gini_{target}∼0.8$) and a growing inequality for drugs ($Gini_{drug}∼0.6$) (Figure S12), highlighting that a few targets and drugs are tested at high rates.

#### Predicting drug exploration

We utilize an Auto-Regressive Integrated Moving Average (ARIMA) time series model^51^ to predict the drug exploration patterns. The model accounts for seasonal variation in trends to forecast future events. We consider the number of new targets tested every year as the output variable and estimate the best model fit using root mean square error estimation (RMSE). We utilize 80% as training data and find the best model fit (1, 0, 3) with RMSE 50 (Figure S11). The model estimates that by 2025, 2,477 targets will be tested (95% confidence interval: 2,445 - 3,682).

#### Impact of approvals and patents

Every investigational drug has an estimated patent period for 20 years, after which the exclusive rights for marketing that molecule expires.^40^ We can then estimate ∼8 years until the drug completes clinical trials, providing about 8 to 13 years post approval until the patent expiry. We find that the number of trials for approved drugs increases rapidly post approval (Figure S14). Yet, the trials post approval test conditions different from the FDA approved condition, also referred to as drug repurposing (Figure S16, indicating that drugs receive increased attention post approval, primarily for diverse diseases.

Finally, we examine the time spent on the FDA approval process. We consider the first completion date of a Phase 3 trial to signal completion of the clinical development. We find that an average drug spends about 3 years after Phase 3 completion to receive approval (Figure S15), suggesting a delayed approval period for each drug.

#### Mapping the clinical exploration trajectory of proteins

The first time a protein is associated with a drug in a clinical trial is an important parameter as it represents the year the scientific community recognized the therapeutic utility of that protein. Similarly, the first approved drug of a protein indicates the protein was associated with a drug that showed promising effects in clinical trials. Since new proteins are rarely selected as targets in clinical trials, we next measured the time span between a protein’s discovery and its emergence as a target in a clinical trial. We find that it takes a protein, on average, 16 years after its discovery to take part in its first clinical trial and takes another 6 years after the first drug targeting it to receive the first approval (Figure S17).

For example, the protein DNMT1, which is associated with dementia, bipolar disorder and some rare diseases such as leukemia, pulmonary fibrosis, was discovered in 1988, and first entered clinical trials as a target seven years later, in 1995 (Figure S18A, top). Its second trial was in 1999, and the third in 2000. In 2004, the first drug that targeted the protein was approved, followed by another drug approved in 2006. Similarly, the protein TPH1, which is associated with multiple mental disorders, was discovered in 1987, and its first clinical trial was in 2004, 17 years after its discovery. The second drug was tested in 2006, and the first approved drug emerged in 2007 (Figure S18A, bottom). These exploration patterns prompted us to introduce two variables to quantify recency: 1) time to first trial since discovery of a protein, and 2) time to first approval since the first trial.

We utilize the Kaplan-Meier survival curves^52^ to estimate the time to event variables. We find that the time to subsequent trials decreases if a protein is targeted by multiple drugs (Figure S18B), indicating that clinical trials are more likely to focus on recently tested targets. That is, the more drugs target the protein, the more experimental validity it receives, decreasing the time until a subsequent trial. In a similar fashion, the time to approval for targets decreases as it becomes associated with several approved drugs (Figure S18C), hence the time to second approval is much shorter than the time to first approval, and so on. In summary, we find that proteins experience a long wait time until their first trial as a target, but recently targeted proteins are more likely to be selected for new drugs.

Further, we find non disease genes enter the trial rapidly after approval but a higher proportion of disease genes eventually receive a trial (Figure S20A). Interestingly, there are no differences in the survival times of common and rare disease genes (log rank test: 0.29, p = 0.58). Further, we find that genes associated with no diseases are less likely to be associated with an approved drug (Figure S20B). Unsurprisingly, druggable genes are more likely to be in a trial and more likely to be approved than non druggable genes (Figures S20C and S20D).

#### Repeated occurrence of proteins

We model the dynamics of repeated occurrences of proteins in trials using the PWP Gap Time model,^53^ a survival model for event recurrence estimation, where the time to event resets based on sequential occurrence of events. Specifically, the proteins are stratified based on the clinical trial events, for example, first drug trial, second drug trial. We find that a target’s hazard ratio (HR) to be associated to a second drug increases after its first drug trial (HR: 0.82, CI:[0.73, 0.93] vs. HR: 1.22, CI:[1.03, 1.45], p < 0.01: Table S1), indicating that a protein experiences increased likelihood of a new drug after its first drug trial. In summary, we find that proteins experience a long wait time until their first trial as a target, but recently targeted proteins receive increased attention, reducing the time to be subsequently tested for new drugs.

#### GLMM model

The data includes measurements where the same target can be used for multiple new drugs over several years, creating repeated and longitudinal observations for the same target. To model these interactions in a temporal fashion, we consider the generalized linear mixed effects model (GLMM) that accounts for fixed and random effects. We use a binomial regression with a logistic link function:

(Equation 1) $$g\left(E\left[Y_{i}\right]\right)=X_{i}*\beta +Z_{i}*U+\gamma \text{,}$$

where $E\left[Y_{i}\right]$ represents the probability of a protein to be selected as a new drug target. $X_{i}$ represents the explanatory variables associated with fixed effects β; $Z_{i}$ represents the parameter associated with random effects on U, quantified as (i) gene observation (ii) year of clinical trial; and γ represents the model residuals. We consider the following fixed effects variables:

(1) association with a common disease (binary)
(2) association with a rare disease (binary)
(3) disease associated protein in the neighborhood (binary)
(4) number of approved drugs at time $t;n_{approved}^{t}$, (count)
(5) number of approved drugs in the neighborhood at time $t;nn_{approved}^{t}$, (count)
(6) number of clinical trials at time $t;n_{ct}^{t}$, (count)
(7) number of clinical trials in the neighborhood at time $t;nn_{ct}^{t-1}$, (count)
(8) number of drugs at time $t;n_{drug}^{t}$, (count)
(9) number of drugs in the neighborhood at time $t;nn_{drug}^{t-1}$, (count)

The parameters of the GLMM were selected after preliminary data analysis. First, we found that a clear distinction in number of trials based on the disease type association, for example, rare diseases are rarely tested (see Figure S2). This prompts us to consider the disease associations of proteins. Second, we found that 1,260 (92%) of all drugs tend to target at least one protein targeted by an approved drug, prompting us to include drug approval parameters. Third, we found that previously tested proteins tend to be repeatedly tested in clinical trials (see Figure S10), prompting us to include number of previously tested drugs and number of clinical trials as parameters for our model. Finally, we found that the majority of the proteins (76%) selected for new drugs tend to interact with proteins that are previously targeted by drugs, prompting us to incorporate the exploration patterns in the local network neighborhood of the protein.

We explored four GLMM models: (a) Model 1 includes disease related variables (association to a common disease, association to a rare disease, and disease prevalence in the local network neighborhood). (b) Model 2 we consider the number of approved drugs associated to the target and the number of approved drugs associated to the target’s local network. (c) Model 3 we consider the role of clinical trials by capturing the number of previously tested clinical trials on the target and the number of previously tested clinical trials in the network neighborhood (d) Model 4 we consider the target disease variables and the number of experimental drugs associated to the target and the number of experimental drugs associated to proteins in the local network neighborhood. In the models 2 to 4, we also include target specific disease variables, allowing us to better disentangle the effects between disease association and clinical trials drug exploration. We consider all targets that were tested in at least one clinical trial in a given year as positive samples (6%), and the remaining targets as negative samples (94%). We show the results in Table S3 and the results when only considering the primary targets in Table S4).

It is important to note that our model does not investigate the mechanisms behind the discovery of new proteins or help explain the interactions between proteins in the network. Instead, our focus is on utilize the PPI network to understand the underlying processes that lead to the exploration of novel targets. It is worth mentioning that our analysis only considers binary versions of the PPI network.

#### Testing for interactions

To investigate the interaction between the two key identifying results in the GLMM model, we incorporate cross interaction variables that consider the association of the target with both common or rare diseases and its previous testing in clinical trials. By including these cross interaction variables, we aim to measure the combined effect of these two factors on the likelihood of a target being selected for a clinical trial.

(1) association with a common disease (binary)
(2) association with a rare disease (binary)
(3) whether the target was previously tested in a clinical trials (binary)
(4) whether the target is associated to a common disease and it is also previously tested (interaction term)
(5) whether the target is associated to a rare disease and it is also previously tested (interaction term)

We provide the results of our analysis in Table S5. Notably, we observe that when a target is associated with a rare disease and has undergone previous testing in a clinical trial, its likelihood of being chosen for a new clinical trial decreases. This finding provides valuable additional insights into the relationship between target disease associations and their impact on the selection of targets for clinical trials.

#### Network-based drug discovery model

We model choices in drug discovery using two parameters, first is parameter *p* that represents the probability of selecting a previously tested protein and second is parameter *q* that represents the probability of selecting a protein part of a previously explored neighborhood. We utilize the entire search space of *p* and *q* to simulate alternative exploration strategies and examine its related benefits for drug discovery. We consider drug exploration from 2011 to 2020 in our simulations, sampling the exact number of proteins tested every year, m(t).

To test the empirical validity of the model, we utilize the resulting distribution of number of drugs per target for each simulation. The distribution characterization how widely proteins are selected as targets for drugs. We utilize the Kolmogorov-Smirnoff distance to measure the maximum difference between the model and the empirical data. As we show in the main text, the model accurately finds this distribution in the preferential attachment (PA) strategy. Yet, we find that the model fails to recreate the observed patterns if we remove preferential selection of drug targets (Figure S26).

#### Predicting potential drug candidates

To validate the model’s ability to identify potential drug targets, we ask the model to identify drug candidates for three autoimmune diseases - Rheumatoid Arthritis (RA), Crohn Disease (CD), and Asthma. We begin by identifying disease proteins associated to each of the three disease that were tested in previous clinical trials. Next, we search the interaction of these proteins and pick untargeted proteins among them, representing proteins that are part of explored neighborhoods. Next, we use the model to select proteins through the three outlined strategies (PA, R, NS), allowing us to rank proteins based on the frequency they are targeted. Finally, the proteins in the network are validated as druggable, based on extensive experimental studies. We use the well curated list of druggable proteins,^27^ to investigate whether the predicted protein has been verified as a potential drug-target, allowing us to measure if the exploration patterns leads to potential druggable outcomes.

We present the prediction result for the breadth of *p* and *q* parameters. Across all three diseases, we find that 70% of the selected targets through the NS strategy are verified as potential drug candidates (Figure S28). Indeed, the current practices (PA) selects targets with high accuracy but does so at the cost of prioritizing previously tested targets. In contrast, we show that a network-based search process can be an effective way to improve drug discovery in under-explored regions of the interactome.

##### Target validation

Additionally, we conduct in-silico studies by searching the predicted results for the network search (NS) strategy. We present the list of identified proteins for RA, CD, and Asthma in Table S6, along with the specific functions of each protein, provided by GeneCards.^54^

The network model is able to find drug candidates in the local network neighborhood of disease-associated proteins. For example, the method selected the protein NLRP3 as a potential drug candidate for RA. NLRP3 interacts with proteins ABCB1, HSP90AA1, CYP3A4, NR1I2, proteins that have been associated to RA and that were previously tested in clinical trials. Indeed, mutations downstream of NLRP3 play an essential role in regulating the inflammasome, identified as a risk factor for inflammatory diseases.^55^ Animal model studies verified that the regulating the over-expression of this gene inhibits the maturation of interleukin-1β (IL-1β), and reduces RA-induced inflammation.^35^ These results indicate that the model is able to predict potential novel drug candidates. The illustrated technique can be used to conduct *in-silico* testing of the model predictions for multiple diseases.

### Quantification and statistical analysis

#### General statistical analysis

R software was used for statistical tests. The tests used, test outcomes, robustness results can be found in the figure legends.

#### Network separation score

As noted in the main text, 797 (38%) experimental proteins serve as a target of an approved drug. Of the experimental proteins without target of an approved drug, 891 (76%) have at least one protein in its local network neighborhood that targets an approved drug, while 274 (23%) are 2° away from an approved target. Using the separation score, we test the hypothesis that FDA approval patterns affects drug exploration. We classify proteins into two categories: proteins that are associated with approved drugs and proteins that are associated with experimental drugs. This distinction allows us to measure the separation between the two groups. We use the separation score,^34^ defined using,

(Equation 2) $$S_{A,B}=d_{A,B}-\frac{d_{A,A}+d_{B,B}}{2}$$,

where $d_{A,B}$ is the normalized shortest distance between two groups defined as,

(Equation 3) $$d_{A,B}=\frac{1}{\left|A\right|}\sum_{a\in A}\forall_{b\in B}D_{a,b}$$

,where $D_{a,b}$, is the shortest distance between two nodes in the network. This formulation allows us to consider *A* to be the group of experimental proteins and *B* to be the group of approved proteins. We create random networks, sampling the exact number of proteins found in sets *A* and *B*, and measure the separation score of the random samples, $S_{A,B}^{r}$ (Figure S24). We then compute the $z_{score}$ using,

(Equation 4) $$z_{score}=\frac{S_{A,B}-\mu_{S_{A,B}^{r}}}{\sigma_{S_{A,B}^{r}}}$$

## References

[1] DiMasi J.A., Grabowski H.G., Hansen R.W. (2016). Innovation in the pharmaceutical industry: new estimates of r&d costs. J. Health Econ..

[2] Khanna I. (2012). Drug discovery in pharmaceutical industry: productivity challenges and trends. Drug Discov. Today.

[3] Food and Drug Administration (2017).

[4] Avorn J., Kesselheim A., Sarpatwari A. (2018). The fda amendments act of 2007—assessing its effects a decade later. N. Engl. J. Med..

[5] Weiland M. (2020). Missing clinical trial data must be made public, federal judge says. Science.

[6] Casassus B. (2021).

[7] Kozlov M. (2022). Nih issues a seismic mandate: share data publicly. Nature.

[8] Zarin D.A., Tse T., Williams R.J., Califf R.M., Ide N.C. (2011). The clinicaltrials. gov results database—update and key issues. N. Engl. J. Med..

[9] Cihoric N., Tsikkinis A., van Rhoon G., Crezee H., Aebersold D.M., Bodis S., Beck M., Nadobny J., Budach V., Wust P., Ghadjar P. (2015). Hyperthermia-related clinical trials on cancer treatment within the clinicaltrials. gov registry. Int. J. Hyperthermia.

[10] Hirsch B.R., Califf R.M., Cheng S.K., Tasneem A., Horton J., Chiswell K., Schulman K.A., Dilts D.M., Abernethy A.P. (2013). Characteristics of oncology clinical trials: insights from a systematic analysis of clinicaltrials. gov. JAMA Intern. Med..

[11] Pasquali S.K., Lam W.K., Chiswell K., Kemper A.R., Li J.S. (2012). Status of the pediatric clinical trials enterprise: an analysis of the us clinicaltrials. gov registry. Pediatrics.

[12] Bell S.A., Smith C.T. (2014). A comparison of interventional clinical trials in rare versus non-rare diseases: an analysis of clinicaltrials. gov. Orphanet J. Rare Dis..

[13] Brady E., Nielsen M.W., Andersen J.P., Oertelt-Prigione S. (2020). Lack of Consideration of Sex and Gender in Clinical Trials for Covid-19. medRxiv.

[14] Kong W.Y., Saber H., Basha M. (2020).

[15] Cao D.-S., Liang Y.Z., Deng Z., Hu Q.N., He M., Xu Q.S., Zhou G.H., Zhang L.X., Deng Z.x., Liu S. (2013). Genome-scale screening of drug-target associations relevant to k i using a chemogenomics approach. PLoS One.

[16] Jacob L., Vert J.-P. (2008). Protein-ligand interaction prediction: an improved chemogenomics approach. Bioinformatics.

[17] Sonawane A.R., Weiss S.T., Glass K., Sharma A. (2019). Network medicine in the age of biomedical big data. Front. Genet..

[18] Loscalzo J. (2017).

[19] Hopkins A.L. (2008). Network pharmacology: the next paradigm in drug discovery. Nat. Chem. Biol..

[20] Chong C.R., Sullivan D.J. (2007). New uses for old drugs. Nature.

[21] Yao L., Evans J.A., Rzhetsky A. (2010). Novel opportunities for computational biology and sociology in drug discovery: corrected paper. Trends Biotechnol..

[22] Vasan K., West J.D. (2021). The hidden influence of communities in collaborative funding of clinical science. R. Soc. Open Sci..

[23] U.S. Food and Drug Administration (2022). https://www.fda.gov/patients/drug-development-process/step-3-clinical-research.

[24] Gates A.J., Gysi D.M., Kellis M., Barabási A.L. (2021). A wealth of discovery built on the Human Genome Project — by the numbers. Nature.

[25] Krieger J., Li D., Papanikolaou D. (2018).

[26] Brown D.G., Wobst H.J., Kapoor A., Kenna L.A., Southall N. (2021). Clinical development times for innovative drugs. Nat. Rev. Drug Discov..

[27] Freshour S.L., Kiwala S., Cotto K.C., Coffman A.C., McMichael J.F., Song J.J., Griffith M., Griffith O.L., Wagner A.H. (2021). Integration of the drug–gene interaction database (dgidb 4.0) with open crowdsource efforts. Nucleic Acids Res..

[28] Li Y., Meng Q., Yang M., Liu D., Hou X., Tang L., Wang X., Lyu Y., Chen X., Liu K. (2019). Current trends in drug metabolism and pharmacokinetics. Acta Pharm. Sin. B.

[29] Thorlund K., Dron L., Park J., Hsu G., Forrest J.I., Mills E.J. (2020). A real-time dashboard of clinical trials for covid-19. Lancet. Digit. Health.

[30] Merton R.K. (1968). The matthew effect in science: The reward and communication systems of science are considered. Science.

[31] Barabási A.-L., Albert R. (1999). Emergence of scaling in random networks. science.

[32] Yildirim M.A., Goh K.I., Cusick M.E., Barabasi A.L., Vidal M. (2007). Drug–target network. Nat. Biotechnol..

[33] Morselli Gysi D., do Valle Í., Zitnik M., Ameli A., Gan X., Varol O., Ghiassian S.D., Patten J.J., Davey R.A., Loscalzo J., Barabási A.L. (2021). Network medicine framework for identifying drug-repurposing opportunities for covid-19. Proc. Natl. Acad. Sci. USA.

[34] Menche J., Sharma A., Kitsak M., Ghiassian S.D., Vidal M., Loscalzo J., Barabási A.L. (2015). Uncovering disease-disease relationships through the incomplete interactome. Science.

[35] Liu P., Wang J., Wen W., Pan T., Chen H., Fu Y., Wang F., Huang J.H., Xu S. (2020). Cinnamaldehyde suppresses nlrp3 derived il-1β via activating succinate/hif-1 in rheumatoid arthritis rats. Int. Immunopharm..

[36] Rzhetsky A., Foster J.G., Foster I.T., Evans J.A. (2015). Choosing experiments to accelerate collective discovery. Proc. Natl. Acad. Sci. USA.

[37] Fortunato S., Bergstrom C.T., Börner K., Evans J.A., Helbing D., Milojević S., Petersen A.M., Radicchi F., Sinatra R., Uzzi B. (2018). Science of science. Science of science Science.

[38] Golec J.H., Vernon J.A. (2007).

[39] Kola I., Landis J. (2004). Can the pharmaceutical industry reduce attrition rates?. Nat. Rev. Drug Discov..

[40] Price W.N. (2020). The cost of novelty. Columbia Law Rev..

[41] Munos B. (2009). Lessons from 60 years of pharmaceutical innovation. Nat. Rev. Drug Discov..

[42] Gagne J.J., Choudhry N.K. (2011). How many “me-too” drugs is too many?. JAMA.

[43] Wertheimer A.I., Santella T.M. (2004).

[44] DiMasi J.A., Faden L.B. (2011). Competitiveness in follow-on drug r&d: a race or imitation?. Nat. Rev. Drug Discov..

[45] Naci H., Carter A.W., Mossialos E. (2015). Why the drug development pipeline is not delivering better medicines. BMJ.

[46] Wishart D.S., Feunang Y.D., Guo A.C., Lo E.J., Marcu A., Grant J.R., Sajed T., Johnson D., Li C., Sayeeda Z. (2018). Drugbank 5.0: a major update to the drugbank database for 2018. Nucleic Acids Res..

[47] Morselli D.M., Do Valle I., Zitnik M., Ameli A., Gan X., Varol O., Ghiassian S.D., Patten J.J., Davey R.A., Loscalzo J. (2021). Network Medicine Framework for Identifying Drug Repurposing Opportunities for Covid-19.. Proc. Natl. Acad. Sci..

[48] Piñero J., Ramírez-Anguita J.M., Saüch-Pitarch J., Ronzano F., Centeno E., Sanz F., Furlong L.I. (2020). The disgenet knowledge platform for disease genomics: 2019 update. Nucleic Acids Res..

[49] Lounkine E., Keiser M.J., Whitebread S., Mikhailov D., Hamon J., Jenkins J.L., Lavan P., Weber E., Doak A.K., Côté S. (2012). Large-scale prediction and testing of drug activity on side-effect targets. Nature.

[50] Rolf M.G., Curwen J.O., Veldman-Jones M., Eberlein C., Wang J., Harmer A., Hellawell C.J., Braddock M. (2015). In vitro pharmacological profiling of r406 identifies molecular targets underlying the clinical effects of fostamatinib. *Pharmacol. research & perspectives***3**.

[51] Box G.E., Jenkins G.M., Reinsel G.C., Ljung G.M. (2015).

[52] Kaplan E.L., Meier P. (1958). Nonparametric estimation from incomplete observations. J. Am. Stat. Assoc..

[53] Prentice R.L., Williams B.J., Peterson A.V. (1981). On the regression analysis of multivariate failure time data. Biometrika.

[54] Safran M., Rosen N., Twik M., BarShir R., Stein T.I., Dahary D., Fishilevich S., Lancet D., Abugessaisa I., Kasukawa T. (2021). Practical Guide to Life Science Databases.

[55] Villani A.-C., Lemire M., Fortin G., Louis E., Silverberg M.S., Collette C., Baba N., Libioulle C., Belaiche J., Bitton A. (2009). Common variants in the nlrp3 region contribute to crohn’s disease susceptibility. Nat. Genet..

